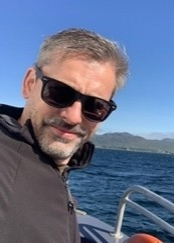# Urea‐Urease Reaction in Controlling Properties of Supramolecular Hydrogels: Pros and Cons

**DOI:** 10.1002/chem.202100490

**Published:** 2021-05-13

**Authors:** Santanu Panja, Dave J. Adams

**Affiliations:** ^1^ School of Chemistry University of Glasgow Glasgow G12 8QQ UK

**Keywords:** Institute and/or researcher Twitter usernames: @prof_djadams, hydrogels, pH-responsiveness, kinetic control, urease-urea reaction, dynamic gels

## Abstract

Supramolecular hydrogels are useful in many areas such as cell culturing, catalysis, sensing, tissue engineering, drug delivery, environmental remediation and optoelectronics. The gels need specific properties for each application. The properties arise from a fibrous network that forms the matrix. A common method to prepare hydrogels is to use a pH change. Most methods result in a sudden pH jump and often lead to gels that are hard to reproduce and control. The urease‐urea reaction can be used to control hydrogel properties by a uniform and controlled pH increase as well as to set up pH cycles. The reaction involves hydrolysis of urea by urease and production of ammonia which increases the pH. The rate of ammonia production can be controlled which can be used to prepare gels with differing properties. Herein, we show how the urease‐urea reaction can be used for the construction of next generation functional materials.

## Graphical abstract

**Autocatalytic reaction** between urease and urea produces ammonia in aqueous medium which increases the pH. Here, we summarize how the urea‐urease reaction can be used to control hydrogel properties. The advantages and limitations of this method compared to conventional methods of pH increase are also discussed.

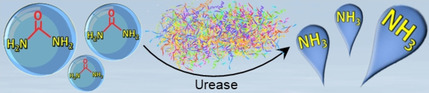

## 1 Introduction

There has been a recent surge of attention into the development of self‐assembled molecules as functional materials.^[1]^ Among various self‐assembled systems, supramolecular hydrogels are fascinating soft materials having multifunctional applications in various fields including optoelectronics and biomaterials.^[2]^ Typically, hydrogels are formed from the self‐assembly of molecules (called gelators) in water under the influence of different non‐covalent forces like hydrogen bonding, π‐stacking, hydrophobic interactions and ionic interactions.^[3]^ These interactions are individually weak. However, when they function in tandem, self‐assembly occurs leading to fibre formation. These fibres entangle or cross‐link to form the underlying gel matrix which immobilizes the water. As a result, despite water being typically ∼99 % of the weight of the gel, these materials behave as viscoelastic solids. Such gels are cheap, can be prepared from easily available building blocks and can have readily tunable properties.

**Figure 1:**
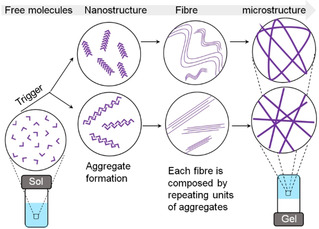
Schematic representation of multilevel self‐assembly during gelation. The assembly kinetics can affect all levels of the assembly. Depending on the trigger, different nanoaggregates can be formed leading to different microstructures and gel properties.

To prepare the gels, the first thing is to activate the molecular building blocks to start the self‐assembly (Figure 1). This is usually achieved by applying a trigger like a pH change, adding a co‐solvent, UV light irradiation, a temperature change, or addition of ionic analytes to a solution or suspension of the molecules. This results in a significant decrease in their solubility.^[3]^ How the activation of the molecules is carried out is important in determining the final properties of the materials. pH responsive hydrogels are common.^[4]^ In these systems, the gelator molecules contain a pH‐responsive functionality. The degree of solubility of the molecules in solution is governed by the degree of ionization (i. e., protonation or deprotonation) of that functional group. pH changes are generally carried out by the addition of a strong acid or base. However, this leads to rapid pH changes, meaning the rate of gelation is often higher than the rate of mixing of the components, leading to inhomogeneous gels with properties that are hard to reproduce and control.^[5]^ Hence, the properties vary depending on the self‐assembly kinetics even though the final composition remains the same. To obtain homogeneous and reproducible gels, it is important to control the gelation conditions.^[5]^

In this context, the urease‐urea reaction provides a means of controlling the properties of pH‐responsive systems. This reaction involves hydrolysis of urea by the enzyme urease producing ammonia, which results in increase in the pH. The rate of the reaction, and so pH change, can be controlled by adjusting the concentration of urease and urea. Although the discovery of the reaction is a century old,^[6]^ exploitation of this method in the field of supramolecular gels has only received attention in last decade. Here, we summarize how the urea‐urease reaction can be used to control hydrogel properties. The advantages and limitations of this method is also addressed, and we discuss how this reaction can be used to construct next generation functional materials.

## 2 Why do we need to control supramolecular gelation?

Formation of the gel matrix from individual molecules occurs through a multilevel self‐assembly process (Figure 1).^[5]^ First, the solubility of gelator decreases in response to the trigger which drives initial aggregate formation (the nanostructure). The intermolecular interactions control the formation of such nanostructures; for gelation to occur these are typically one‐dimensional anisotropic fibres. These fibres subsequently entangle into a three‐dimensional cross‐linked network, immobilising the solvent through capillary forces and surface tension. The cross‐links are also noncovalent in nature, which means that the gels are reversible and so return to the solution state when a counter‐trigger is applied.

The properties of the gels arise from the underlying network structures formed during the self‐assembly process. The average fibre thickness, degree of branching or crosslinking, distance between the crosslinking points as well the nature of the distribution of fibres at a larger length scale (the microstructure) are all determining factors of the gel properties.^[5]^ Since, the solubility of molecules decreases in presence of the trigger, gelation can be considered as a process where the system goes from a ‘highly‐soluble’ environment to a ‘poorly‐soluble’ environment. Hence, gelation is strongly kinetically dependent meaning that the nanostructure formation followed by growth of fibres, as well as distribution of fibres are all highly controlled by the rate of gelation. In short, the microstructure of the gel phase is very dependent on the self‐assembly kinetics.^[5]^

**Figure 2:**
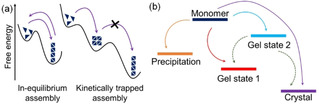
(a) Schematic representation of possible energy landscapes during the self‐assembly of supramolecular materials. Gelation can be considered of as a kinetically trapped state, rather than the thermodynamic minimum. (b) The process of assembly may result in different pathways being followed.

Ideally, when the conditions are changed to induce self‐assembly, there should be a sufficient number of free molecules present in solution so that an exchange of building blocks from solution to the assembled structure is possible allowing the system to reach a low energy state (Figure 2).^[7]^ If any errors are present in the assembled structure, the exchange of molecules ensures rapid correction to attain the more favourable thermodynamically minimum state. In case of gelation, typically, the self‐assembly process occurs at a high rate meaning that homogeneous mixing of components is often impossible. The self‐assembled structures do not have time to reach their optimal global thermodynamic minimum and so exist in a kinetically‐trapped state (a local thermodynamic minimum).^[7]^ The final properties of the gels therefore significantly depend upon the preparative pathway and hard to reproduce.

During self‐assembly, the molecules condense, and it is also possible that the system leads to precipitation and crystallization. While a random aggregation produces precipitation, a highly ordered assembly give rise to crystals. An assembly process intermediate between these two situations results a gel.^[8]^ However, the results are unpredictable and vary from system to system. For instance, Baral *et al*. reported that fast cooling of a peptide solution produced a kinetically‐trapped gel, but significantly slower cooling triggered the formation of turbid solution.^[9]^ Although for both gelation and crystallization, an ordered network structure is needed, we have argued that the three‐dimensional molecular packing in a gel is less ordered than crystals.^[10]^ Crystallization of a gelator from the gel phase is possible indicating that the crystal state is thermodynamically more stable and the gel phase is kinetically trapped.^[11]^ Again, this highlights the need to control kinetics during gelation to avoid competing pathways.

## 3 pH‐triggered hydrogels

pH is one of the most common triggers used to form supramolecular hydrogels.^[4b]^ pH triggered hydrogels are formed by gelators containing pH dependent ionizable functional units such as carboxylic acids, sulphonic acids, pyridines, amines etc. where the solubility of the molecules can be controlled by the degree of protonation or deprotonation of the groups. The mechanism of gelation involves either acceptance or release of protons by these functional moieties in response to a change in the environmental pH. Typically, the gelator solution is prepared first by dissolving in water in presence of acid or base (depending on the functional group). Ionization of the functional group occurs which results in an increase in the electrostatic repulsion between the molecules and so dissolution or at least suspension of the molecules. Gel formation occurs when the pH of the solution is altered by adding base or acid as suitable. The electrostatic repulsion decreases due to reverse ionization, solubility decreases and non‐covalent interactions between the building blocks result in self‐assembly.

Depending upon the ionizable functionality, the gelators can be classified as acid‐triggered or base‐triggered. Acid‐triggered gels are often prepared by adding mineral acids like HCl, AcOH etc. to the alkaline solution of the gelators that brings about a sudden decrease of pH. Homogeneous mixing of the components is difficult here as the rate of gelation is often higher than the rate of mixing. As a result, inhomogeneous hydrogels are often formed.^[12]^ For example, in the case of acid‐triggered Fmoc‐dipeptide gels, mixing is a real issue; the inhomogeneities in the gels can be seen by eye.^[12]^ Similarly, Helen *et al*. reported that the gel properties depends heavily on the mixing rate.^[13]^ To overcome this, our group introduced hydrolysis of glucono‐δ‐lactone (GdL) to gluconic acid as a method of slow pH decrease that lead to significantly more homogeneous gels.^[12]^

## 4 Conventional methods to prepare base‐triggered hydrogels

When considering base‐triggered hydrogels, aggregation is induced by increasing the pH of the gelator solution from acidic to basic. Metal hydroxide solutions (such as NaOH or KOH) are mostly used to drive the pH change.^[14]^ Another possibility is the addition of relatively weak base like NH_4_OH.^[14]^ Basic buffers such as phosphate buffer saline and sodium carbonate buffer are also used to increase the pH.^[15]^ Again, simple addition of aliquots of base or buffer solution can induce catastrophic changes in the medium.^[14]^ Diffusion of gaseous ammonia (ammonium hydroxide vapor) into the gelator solution has been employed to prepare gels.^[16]^ In this case, it is also difficult to prepare homogenous gels as there may be pH gradients within the system since gelation begins from the gas‐liquid interface. There is also a limitation to the volume of gel that can be prepared. Apart from these examples, Nakanishi and co‐workers utilized pyrolysis of urea in water to generate ammonia in situ to prepare silica aerogels.^[17]^ Again, the volume of the gel imposes a limitation here. The requirement to heat the system is another limitation.

## 5 The urea‐urease autocatalytic reaction

**Figure 3:**
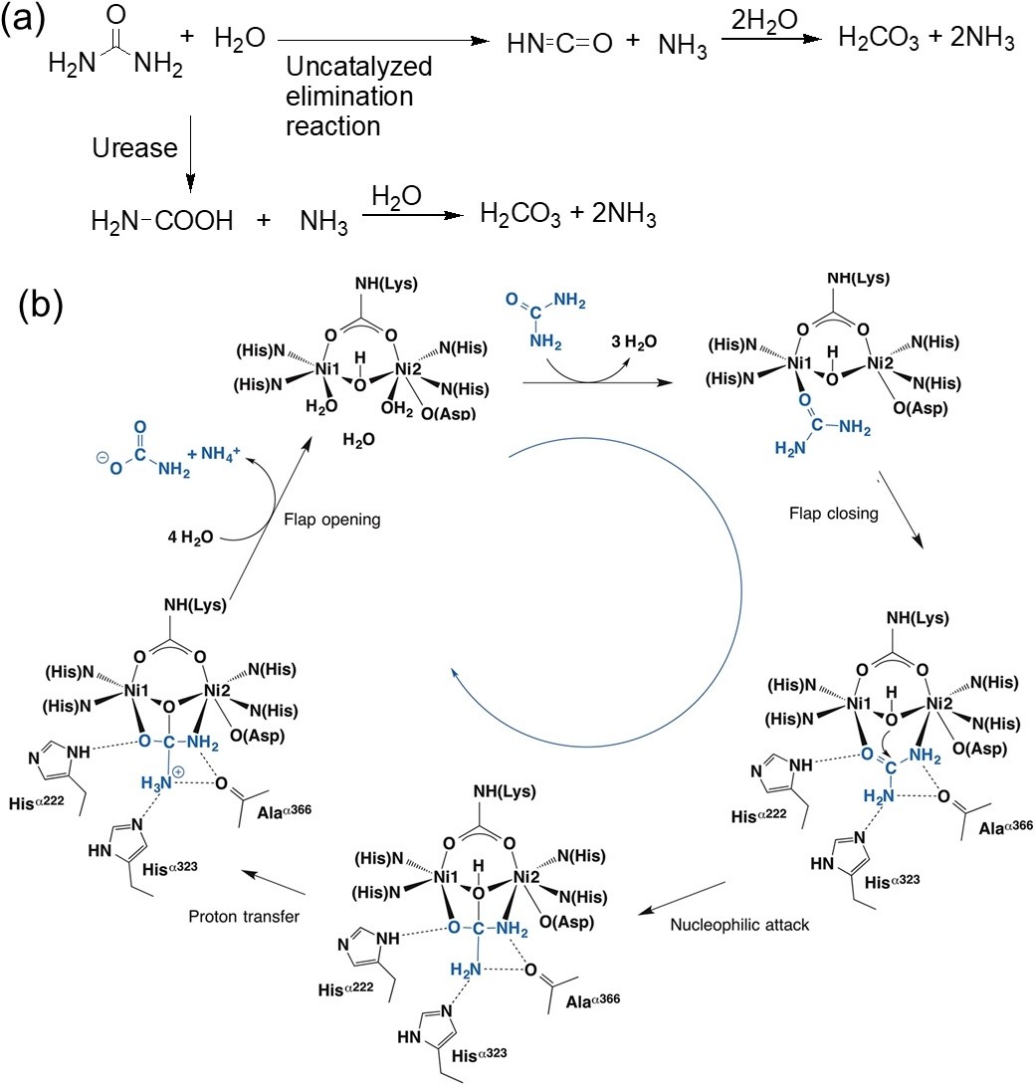
(a) Different modes of hydrolysis of urea. (b) Structure‐based urease catalytic mechanism of the enzymatic hydrolysis of urea. Figure (b) is adapted with permission from Ref. [6]. Copyright © 2018 Elsevier.

In water, hydrolysis of urea generates isocyanate and ammonia whilst the former further hydrolyses to another molecule of ammonia and carbonic acid.^[18]^ The reaction typically follows an elimination pathway at a rate independent of pH between pH 2–12.^[18]^ However, owing to high resonance energy of urea (30–40 kcal/mol), the hydrolysis rate is very slow. Urease (urea amidohydrolase, EC 3.5.1.5) is a naturally occurring enzyme that accelerates the hydrolysis reaction by a factor of at least 10^14^ compared to the uncatalyzed reaction.^[18]^ The urease‐urea reaction involves a different mechanism where urea first hydrolyses into ammonia and ammonium carbamate (Figure 3). The ammonium carbamate further hydrolyses to produce another ammonia molecule and carbon dioxide.

Urease is a naturally occurring enzyme produced by plants, bacteria and fungi, but not by animals.^[6]^ The first ureolytic enzyme was isolated by Musculus in 1874 from putrid urine and named by Miquel in 1890. In 1909, Takeuchi discovered urease from soybean (*Glycine max*) providing a plentiful source. In 1926, Sumner crystallized urease from jack bean (*Canavalia ensiformis*) and established the proteinaceous nature of the enzyme for the first time.^[6]^ Dixon et al. revealed the presence of nickel ion (Ni^2+^) in the active site of jack bean urease.^[6]^ The mechanism of the urease‐urea reaction typically involves binding of urea to Ni^2+^ through the carbonyl oxygen (Figure 3).^[6]^ This makes the urea carbon more electrophilic and more susceptible to nucleophilic attack by water. Urea then forms a bidentate bond by coordinating to a second Ni^2+^ ion involving one of its amino nitrogen atoms. Water attack on the carbonyl carbon of urea then results in a tetrahedral intermediate from which NH_3_ and carbamate are released. Formation of ammonia results in increase in pH of the medium that accelerates the decomposition of the carbamate into another ammonia molecule and carbon dioxide further. Hence the reaction is autocatalytic.^[19]^

**Figure 4:**
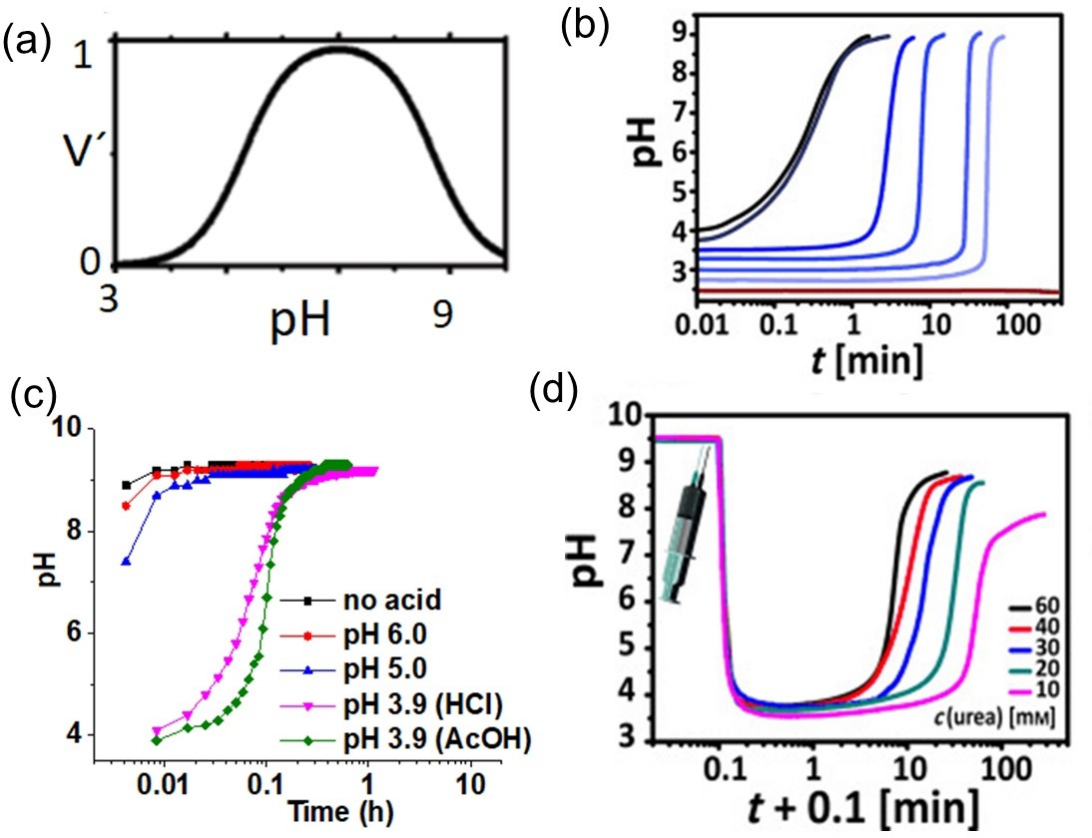
(a) Bell‐shaped pH‐dependent urease activity with a maximum enzyme rate at pH 7. (b) Dependency of urea‐urease reaction on the pH of the medium. The initial pH value controls the enzyme activity and the rate of ammonia production. (c) Influence of the nature of acids on the pH‐time profile. (d) Influence of the initial urea concentration on the rate of pH change as well as on the final pH. Figure (a) is reproduced with permission from Ref. [20a]. Copyright © 2010, American Chemical Society. Figures (b) and (d) are adapted with permission from Ref. [20b]. Copyright © 2015 John Wiley and Sons. Figure (c) is adapted with permission from Ref. [14]. Copyright © 2019 The Royal Society of Chemistry.

There are several advantages of the urea‐urease reaction compared to conventional methods of pH increase. First, the reaction occurs at ambient temperature and hence there is no issue with solvent loss and the volume of gels to be prepared. Second, the reaction is slow, avoiding mixing issues during gelation. Third, the activity of urease is highly pH dependent. The rate vs pH curves are bell‐shaped with a maximum production of NH_3_ at pH 7 (Figure 4a).^[20]^ The enzymatic activity decreases with a decrease in the initial pH of the solution. If the initial pH is sufficiently low (pH<4), the pH‐time profile exhibits a sigmoidal curve where at the beginning the production of NH_3_ is slow but after certain time a rapid conversion to the high pH state occurs (Figure 4b).^[20b]^ Hence, the initial stages of the urea‐urease reaction can show a lag‐phase or induction time if the pH of the medium is acidic.

The lag‐phase can be controlled in a number of ways. The lag‐phase depends on the nature of the acid used for the adjustment of the initial pH (Figure 4c). In case of strong acids such as HCl or H_2_SO_4_, the pH increase is faster compared to weak acid like AcOH.[^14^, ^20a^] Initially, the produced ammonia undergoes salt formation by neutralizing the acid. When a weak acid is used, the produced salt can form a buffer which resists the pH change. For example, in case of AcOH, the rate of pH increase is slow between pH 4 and 5 due to formation of acetic acid‐ammonium acetate buffer (Figure 4c).^[20a]^ Consequently, the lag‐phase increases which can be further extended by increasing initial concentration of the weak acid due to formation of stronger buffer with high acid concentration.[^14^, ^20a^] Due to the catalytic nature, the reaction shows a dependency on the initial concentrations of urea, urease, and temperature.[^20a^, ^21^] At a particular pH, either a decrease in urea or urease concentration, results in a reduction in the rate of ammonia production and thereby increases the lag‐phase (Figure 4d).^[20a]^ Hence, by adjusting the initial conditions one can control the feedback (rate of change of pH) from the urea‐urease reaction. This also influences the final (or maximum) pH of the medium. Typically, the enzymatic reaction can be used to achieve a final pH of ∼9.3–9.5.[^14^, ^20b^] The final pH cannot exceed this due to formation of ammonia‐ammonium buffer at high pH.^[20a]^ However, the final pH strongly depends on initial composition and decreases with decreasing initial urea or urease concentration and increasing initial acid concentration (Figure 4d).^[20a]^ The final pH of the gel is important particularly with the Fmoc‐based gelators since at high pH (typically >pH 10.5), the Fmoc‐group might be deprotected.^[22]^ Fmoc‐derivatives are widely studied in gel chemistry^[23]^ and hence to avoid any stability issue at higher pH it is important to target the final pH lower than 10.5.^[14]^

The nature of the solvent also influences the enzyme activity. Many gels are prepared in semi‐aqueous solvents. In case of mixed solvent systems, presence of polar solvents like *N*,*N*‐dimethylformamide, *N*‐methylformamide etc. are reported to reduces the catalytic activity of urease due to the interaction between the enzyme and the solvent molecules.^[24]^ However, in our recent studies, we did not observe any considerable change in the rate of ammonia production by the urease when the solvent was changed to DMSO/H_2_O (20/80, v/v) from normal water.^[25]^

## 6 Maintaining homogeneity during a sol‐to‐gel transition

**Figure 5:**
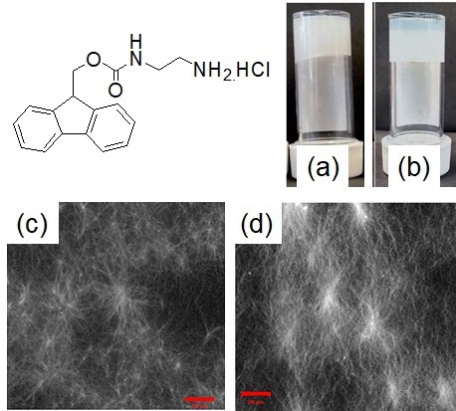
Photographs of hydrogels of the Fmoc‐gelator formed by (a) NaOH and (b) urea‐urease reaction. Confocal microscopy images of the hydrogels from (c) NaOH and (d) urea‐urease reaction (scale bars represent 20 mm). Reproduced with permission from Ref. [14]. Copyright © 2019 The Royal Society of Chemistry.

To obtain gels at high pH, the gelator design is important. Typically, gelators containing pyridine, or amine functional groups are poorly soluble at high pH, and so are widely used to prepare base‐triggered gels. Recently, we utilized the urea‐urease reaction to prepare homogeneous hydrogels from a Fmoc‐based amino compound (Figure 5).^[14]^

The hydrochloride salt of the gelator is soluble in water giving a pH of around 5.1–5.3. Addition of NaOH resulted in formation of the corresponding hydrophobic amine. The pH jump was instant, and gelation began immediately after addition of NaOH yielding a turbid inhomogeneous gel. In comparison, when we used the urea‐urease reaction to change pH of the solution, the rheological changes were consistent with a slow gelation driven by the enzymatic reaction. The time before gelation could be delayed by decreasing the urease concentration or by reducing the initial pH. The enzyme‐triggered gels were translucent as compared to the more turbid gels formed using NaOH because the nature of the underlying fibres of the gels was greatly affected by the gelation kinetics. While the NaOH‐triggered gels exhibited a higher density of spherulitic nucleation domains, the slow enzymatic reaction resulted in a gels with a higher density of long fibres.

**Figure 6:**
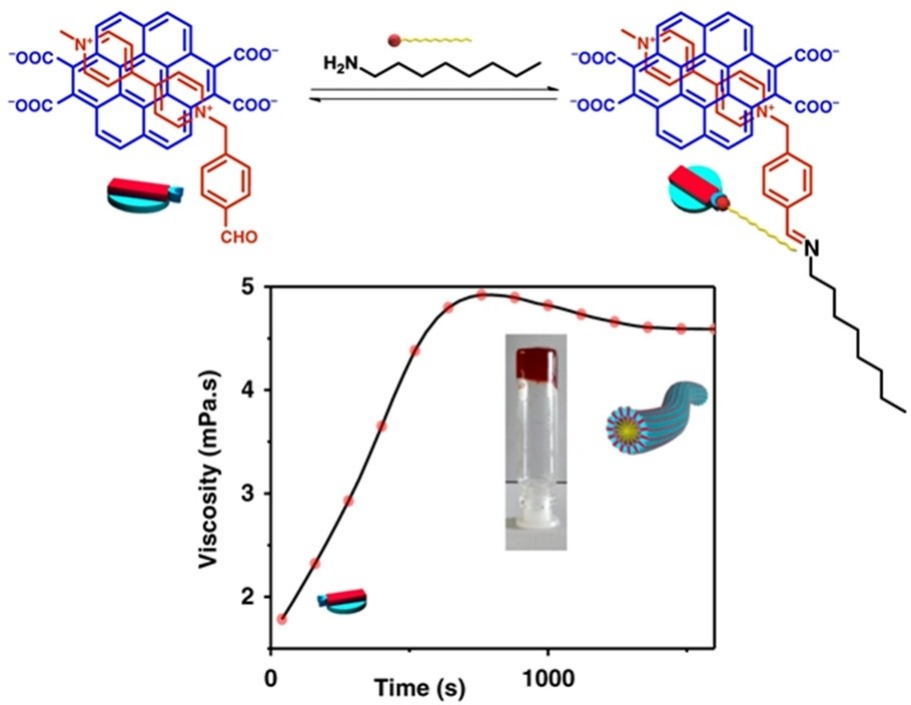
Supramolecular polymerization through dynamic imine bond formation catalysed by urea‐urease reaction. An increase in viscosity indicates immobilization of solvent and gel formation. Reproduced with permission from Ref. [26]. Copyright © 2019, Springer Nature.

George and co‐workers utilized the same reaction to prepare hydrogels involving dynamic imine bond formation of a charge transfer complex (Figure 6).^[26]^ Dynamic covalent bonds are formed or broken reversibly under mild reaction conditions. While formation of an imine bond from the condensation between an aldehyde and an amine is catalyzed at high pH, under acidic condition it undergoes hydrolysis to regenerate the starting precursors. Initially, they prepared a charge transfer complex comprising of a tetra‐potassium coronene salt as a donor and a benzaldehyde substituted viologen as an acceptor which did not have any ability to self‐assembly. On addition of an alkyl amine in the presence of urea and urease, imine bond formation occurred with a gradual pH increase. The charge transfer complex then self‐assembled to form a gel. However, due to poor solubility of the tetra‐potassium coronene salt below pH 7 in water, they set up the initial conditions at pH 7 which led to significant loss of the induction time.

## 7 Recent trends: Temporally programmed dynamic hydrogels

Natural systems exhibit autonomous dynamics and undergo various structural and mechanical changes in response to a change in the environment. Adaptation of living systems by synthetic analogues not only enables to understand fundamental principles of biology but also allows to create life‐like materials with useful and exciting properties.^[27]^ As discussed, most synthetic gels are formed under equilibrium conditions or exist in a deep well of a kinetically trapped state. As a result, they usually do not exhibit changes in material properties with time after formation. However, as the gel structure is maintained by reversible non‐covalent interactions, such systems have the ability to change their properties on perturbation.^[4b]^ The issue is that such switching of properties always demands an outside trigger meaning that self‐regulating and autonomous behaviour is largely absent in traditional synthetic gels.^[27]^

**Figure 7:**
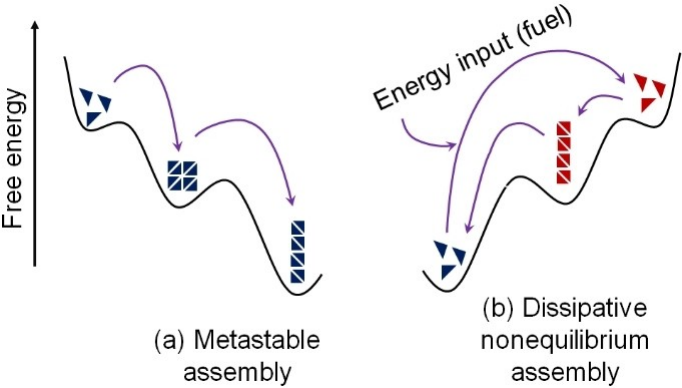
Schematic representation of possible energy landscapes during dynamic evolution of supramolecular gels.

Temporally programmed dynamic hydrogels represent a special class of gel which show time variable changes in their properties. Initially, a gel is formed which either evolves to another gel system or reverts to the solution state. The dynamic nature is governed by the internal mechanism of the system and no external trigger is needed. Incorporation of autonomous dynamic behaviour into traditional gels allows one to prepare materials that cannot be obtained under normal conditions. Before discussing how to do this, it is necessary to understand the difference between the energy landscapes of static and dynamic equilibria. The energy profile diagram for classical/traditional gels is described in Figure 2 where the self‐assembly occurs under kinetic trapping due to lack of exchange of building block between solution and the assembly state. Instead of a global thermodynamic minimum, the structure is trapped in a local thermodynamic minimum from which it cannot escape and no further rearrangement occurs over their useful lifetime.^[7]^ However, in some cases, the exchange of building blocks is possible and a gradual transformation of a kinetically trapped state into a thermodynamically more stable state occurs (Figure 7a). Such systems are referred to as metastable.^[7]^ Formation of metastable materials is governed by both kinetics and thermodynamics where a kinetic product formed first that slowly transforms into a thermodynamic one.^[7]^

Another variation of dynamic assembly transition is if the self‐assembly occurs under thermodynamically unfavourable conditions (Figure 7b).^[7]^ Here, assemblies are formed in presence of an energy input or trigger, the so‐called fuel.^[28]^ However, the precursor molecules are thermodynamically more stable than the assembled structure and so the assembled structure reverts back to the precursor unless a constant influx of fuel is provided. The formation and stability of the assembly is governed by the rate of fuel consumption.

**Figure 8:**
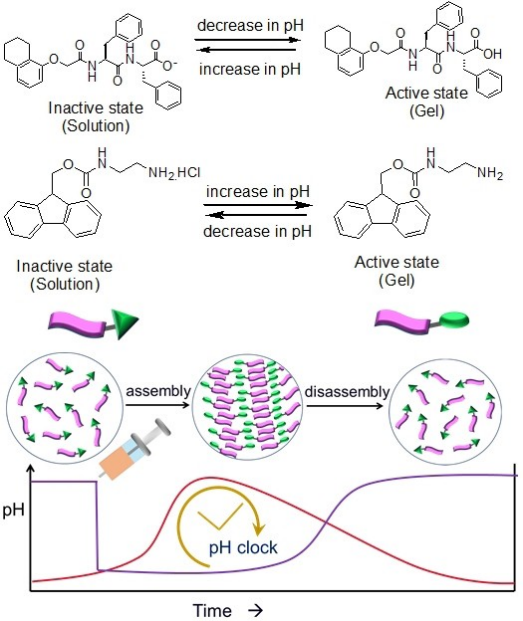
Illustration of pH responsiveness of either a carboxylic acid‐terminated or an amine functionalized gelator along with transient hydrogel formation in presence of acid‐triggered (purple) and base‐triggered (red) pH cycles, respectively. These two compounds are chosen as model examples. For acid‐triggered pH cycle, initially a sudden pH drop occurs due to addition of acid. For base triggered pH cycles the pH decreases with time due to production of acids involving hydrolysis reaction at high pH. Since carboxylic acid and amine group display opposite ionization, for transient hydrogelation, the appropriateness of the pH‐cycle depends on the choice of gelator.

Due to the strong pH dependency, the urea‐urease reaction can be used to synthesise pH‐responsive dynamic hydrogels (Figure 8). Typically, two types of pH regulating pathways can be prepared from this enzymatic reaction. The unidirectional pH change from acidic to basic pH is the simplest pathway, but it is also possible to construct pH cycles. As the enzyme activity is suppressed at a pH of below pH 4 and above pH 9 (Figure 4),^[20a]^ two types of pH cycles are possible. In a case where the enzymatic reaction (starting from an acidic pH) is coupled with a second chemical reaction that decreases the pH, a pH cycle from acidic‐to‐basic‐to‐acidic pH can be fabricated provided that, at the beginning, the rate of ammonia production is higher than the rate of production of the acid. This type of pH cycle is categorized as a base‐triggered pH cycle; production of base drives the pH decrease. Generally, hydrolysing agents like GdL and methyl formate are used to construct such pH cycle as, at low pH, their hydrolysis rate is low but is high at high pH. In comparison, if the high pH solution of the enzyme (pH 9) is treated with a sufficiently acidic solution of urea, initially it will cause a rapid pH drop followed by gradual increase in pH due to production of ammonia. This pH cycle is acid‐triggered as an acid‐induced pH drop initiates the urea‐urease reaction. The pH cycle changes from basic‐to‐acidic‐to‐basic. In both cases, the first step is the activation step while the second is the deactivation step. When coupling gelators with these possible pH‐changing pathways, the systems respond in two different ways. An initially formed hydrogel may undergo sol formation and so exhibits transient gel behaviour. Alternatively, the gel can evolve to a different gel with time.

### 7.1 Transient hydrogels

The design and synthesis of transient hydrogels has gained tremendous recent interest.[^7^, ^29^] Transient systems allow temporal and repetitive accessing of gel states that cannot be obtained under normal conditions. To form transient gels, the rate of assembly formation should be initially higher than the rate of deactivation. The overall concept is to create active structural elements that self‐assemble in an active environment (i. e., self‐regulating pH states) whilst control over the rate of assembly and disassembly kinetics allows temporal programming of the hydrogel properties.

Transient hydrogels are useful in many ways.^[28]^ They can be explored for on demand applications, burst release of encapsulated materials, self‐erasable and rewriting display materials etc. Another advantage of such gels is that they can be re‐used after re‐fuelling. Such gels have ability to complete multiple cycles without the degradation of the system components. However, it has been observed that on repetitive cycles, the performance of the gel diminished, i. e., the lifetime as well as mechanical properties changed.[^7^, ^30^]

Both acid and base triggered pH cycles are used to construct transient hydrogels. However, the choice of the pH cycle entirely depends on the pH‐responsive ionization of the gelators. For example, carboxylic acid functionalized peptide gels are stable at acidic pH while at basic pH, due to carboxylate formation, the gels collapse. Hence, acid‐triggered pH cycles are generally employed on peptide gelators to construct transient hydrogels at low pH (Figure 8). Similarly, base‐triggered pH cycles are effective to drive transient hydrogelation of amines at high pH.

The Walther group synthesized a time‐programmed transient hydrogel system by combining the urease‐urea switch with an acidic buffer (citric acid/sodium citrate buffer) involving a Fmoc‐dipeptide.^[20b]^ Initially, due to rapid pH decrease, an acidic state was created to form a gel. Subsequently, increased enzyme activity promoted the generation of alkaline ammonia and drove the disassembly. The duration of the transient acidic profile was controlled by varying the urease concentration or the buffer strength which enabled the lifetime of the gel to be tuned from a few minutes to several hours. The self‐regulating hydrogels were exploited to design self‐erasing ink, temporally block microfluidic channels and reroute fluid flow in a simplistic vascular network model in a time pre‐programmed fashion. They further employed the pH cycle in a polymer gel, by which the reflective state was programmed.^[31]^ The photonic gel films were comprised of pH‐sensitive polystyrene‐*b*‐poly(2‐vinyl pyridine) (PS‐*b*‐P2VP) block copolymers. At a pH below the p*K*_a_, the protonation of pyridine groups of the polymer resulted in an expanded volume, producing a photonic band gap in the visible region. When the pH was raised up to pH 4.5, the deprotonation occurred which caused volume contraction. On integration of the urea‐urease pH‐switch, a time programmable photonic display with different lifetimes was accomplished by altering different enzyme concentrations.

Following a similar concept, Mondal *et al*. reported an acid‐triggered transient hydrogelation of benzyloxycarbonyl‐L‐phenylalanine. The only difference in the pH cycle is that HCl was used to induce the pH drop. They found that while the peptide fibres exhibited positive results (green‐gold birefringence under polarized light) in the Congo red assay, the solution obtained after the gel‐to‐sol transition generated negative response. From this study, it was concluded that the pH acted as a trigger for the amyloid‐like assembly of phenylalanine.^[32]^

**Figure 9:**
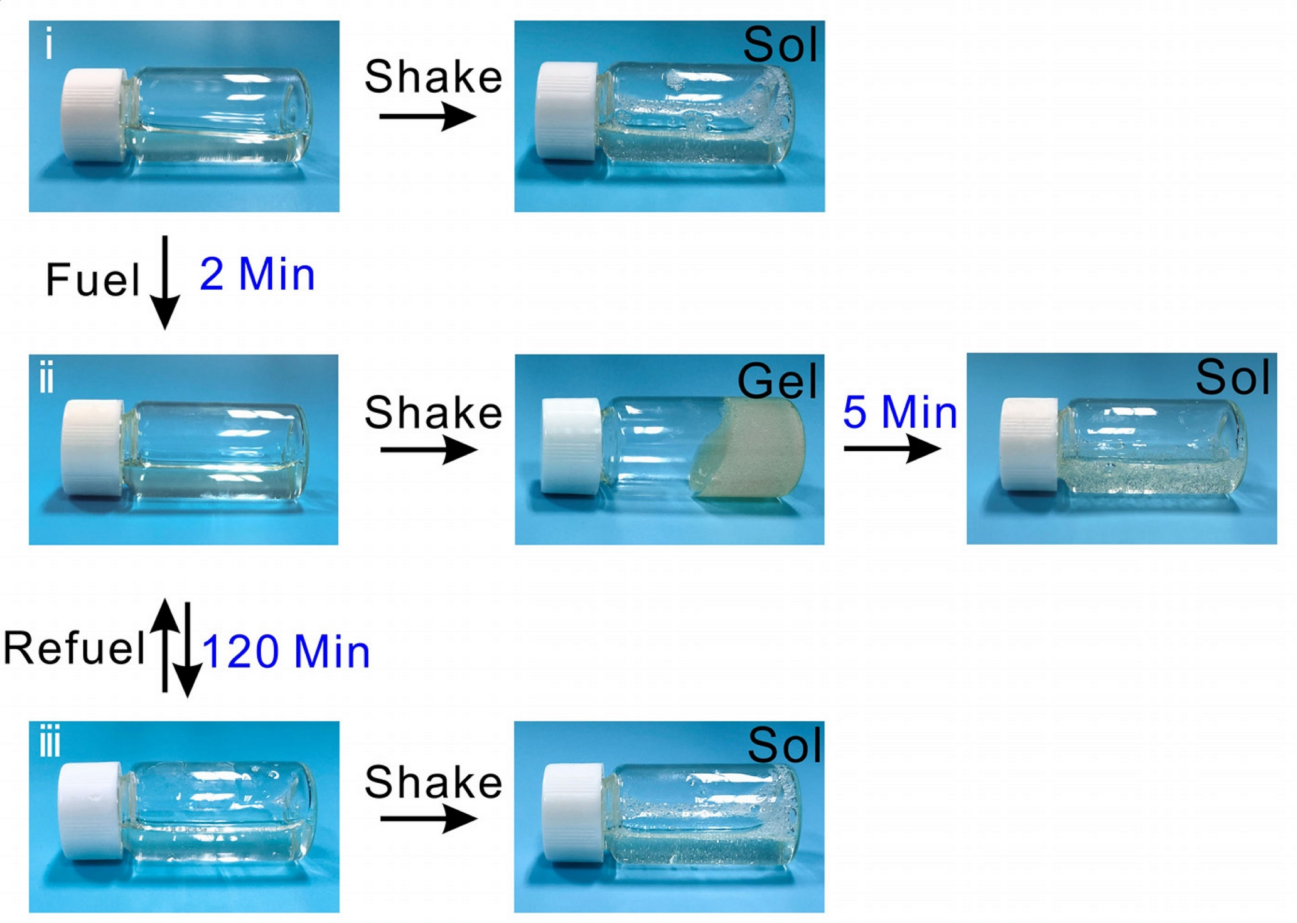
Chemical fuel‐controlled field‐responsive fluidic materials developed by Yang group.^[33]^ a) Photographs representing the time‐dependent non‐Newtonian behavior of fluid: i no shake‐gel before fuel addition, ii) transient shake‐gel behavior after fuel addition, iii) recovery of initial shake behavior after the energy dissipation with time. Adapted with permission from Ref. [33]. Copyright © 2020 John Wiley and Sons.

Recently, the Yang group utilized the biocatalytic feedback of the urea‐urease reaction to temporarily program the properties of non‐Newtonian polymer gels.^[33]^ Several pH‐responsive polyelectrolytes were synthesized by the polymerization of *N*,*N*‐dimethylacrylamide (DMA) and *N*,*N*‐dimethylaminoethylmethacrylate (DMAEMA), followed by hydrophobic modification and urease immobilization. At a pH of 7.5 or higher, the deprotonated associative polyelectrolytes formed compact coil structures in solution, exhibited a shear thinning state. As the pH decreased, protonation of the tertiary amine groups led to coil expansion and resulted in an unexpected shear gelling phenomenon. Coupling of the urease‐urease reaction resulted in switching between these two non‐Newtonian states. As can be seen from Figure 9, the initial polymer solution (pH∼9.0) maintained its flowing, liquid‐like property whether at rest or being shaken. When a urea‐containing acidic buffer (the fuel) was added, the acidic fluid showed a “shake‐gel” behaviour i. e., the shear thickening state. The immobilized urease polymer gradually converted the urea into ammonia. Subsequent pH increase resulted in deprotonation and enabled the system to recover the original shear thinning response.

The Walther group prepared a pH‐responsive transient DNA hydrogel.^[34]^ They used ethyl acetate as hydrolysing agent and incorporated an esterase enzyme into system to control the rate of pH decrease. The esterase enzyme has a low activity at low pH and an almost constant plateau at pH>6. The aim was to activate the esterase through the pH change caused by the urea‐urease reaction and thereby to control the assembly and disassembly kinetics. We used a base‐triggered pH cycle to create a temporary high pH state and thereby to programme the hydrogel properties at high pH.^[30]^ We combined the urease‐urea reaction with methyl formate to construct the pH cycle in presence of a hydrochloride salt of a Fmoc‐based amine. An initial free flowing solution became a gel by a gradual increase in pH. After certain time, the hydrolysis of methyl formate became predominant and the production of acid caused a pH decrease. The gel eventually collapsed and finally reverted to the initial solution state. Confocal microscopy showed that aggregation begins within 2 minutes with the appearance of spherulitic domains of fibres. The density of the spherulitic structures increased with time and interlinked fibres were found at high pH. As the pH then decreased, the fibres started to disintegrate into discrete spherical aggregates and disappeared after 15 hours. Variation of gelator concentration, volume of methyl formate as well as concentrations of urease and urea allowed controlling of the gel lifetime from few minutes to several hours.

**Figure 10:**
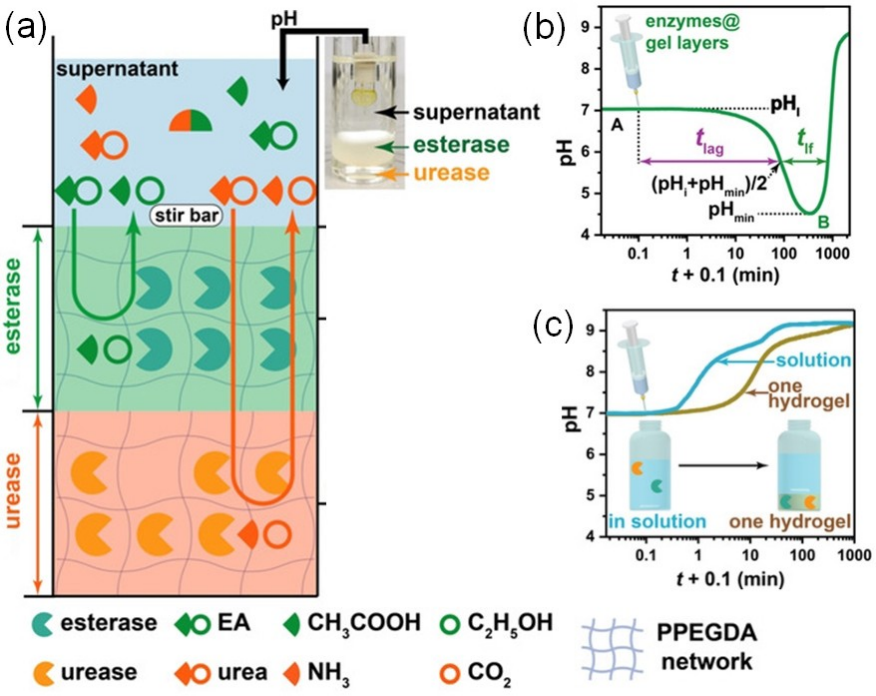
(a) Demonstration of the transient acidic pH flip involving a tri‐layered system. While esterase embedded gel layer (middle) catalyzed the hydrolysis of ethyl acetate to acetic acid, urease encapsulated gel layer (bottom) converted urea to NH_3_ and CO_2_. (b) Change of pH with time in the supernatant layer (top) involving the tri‐layered system described in (a). (c) Change of pH with time in the supernatant layer when both the enzymes were encapsulated in a single gel layer. Reproduced with permission from Ref. [35]. Copyright © 2020 John Wiley and Sons.

In a recent study, the Walther group demonstrated autonomous transient pH flips by spatial compartmentalization of two antagonistic enzymes and thereby to control lifetime of transient aggregates.^[35]^ They used a tri‐layered system (Figure 10); the bottom and middle layers contained urease and esterase enzymes respectively encapsulated in a photo‐crosslinked poly(poly(ethylene glycol) diacrylate) gel. The top layer was a supernatant layer to which various fuels like urea and ethyl acetate, buffer and gelators were added. When the fuels (urea and ethyl acetate) were injected, they were converted to ammonia and acetic acid due to hydrolysis by urease and esterase respectively followed by diffusion of these components into the supernatant which controlled the pH‐time profile. As the esterase layer was on top of the urease layer, initially a pH drop was noticed due to faster production of acetic acid. With time, the production of ammonia starts which slowly diffuse into the supernatant layer and raised the pH to basic. A transient acidic pH flip was achieved by this way whilst incorporation of a high pH peptide solution to the supernatant layer resulted in a transient aggregation. When the enzymatic gel layers were inverted, an opposite alkaline pH flip was achieved which was utilized to fabricate a transient assembled state of an Fmoc‐amine. At higher fuel concentrations, the transient aggregates eventually produced a transient gel. In both cases, the lag phase, depth/height of the pH jump, lifetime of the transient pH states as well as final pH of the supernatant solutions could be controlled by varying the height of the layers, enzymes and fuel concentrations. Such transient pH flips are not possible if both the enzymes are encapsulated into a single gel layer. The integration of the three‐layer system not only allowed temporal domains but also enables spatial programming of material properties.

### 7.2 Dynamic gel‐to‐gel transitions

Another variation of self‐regulating dynamic gels is where there is a reconfiguration of state such that there is a change in underlying structure from one gel to a second, different gel. Such dynamic gel‐to‐gel transitions can be used to prepare materials with adaptive properties. Unidirectional pH increases as well as pH cycles can both be used. Typically, the lag phase associated with the enzymatic reaction is used to drive changes to a primary assembled state formed by a fast activation process. For example, we used a solvent trigger to form gels almost instantly using a peptide gelator.^[36]^ The hydrogels had a pH of around 4.1. Coupling of the urea‐urease reaction with this hydrogel resulted in a gel‐to‐sol transition with a pH rise to pH 9. When the same reaction was performed in presence of Ca^2+^, the gel at low pH formed a sol phase as the pH increased, followed by Ca^2+^ ions binding to the carboxylates of micellar structures at high pH which caused reappearance of the gel. Time sweep rheology and confocal fluorescence microscopy confirmed the gel‐to‐sol‐to‐gel transition. The lifetime of the initial gel as well as mechanical properties of the final gel were both strongly dependent on the rate of pH increase. The pH change could be controlled by adjusting the initial concentrations of urease, urea and Ca^2+^ ions. The high pH Ca^2+^‐triggered gels were transparent. whilst direct hydrogelation by adding Ca^2+^ ions to the alkaline solution led to turbid inhomogeneous gels, showing the enzymatic approach is effective in inducing homogeneous hydrogelation of peptides at high pH.

**Figure 11:**
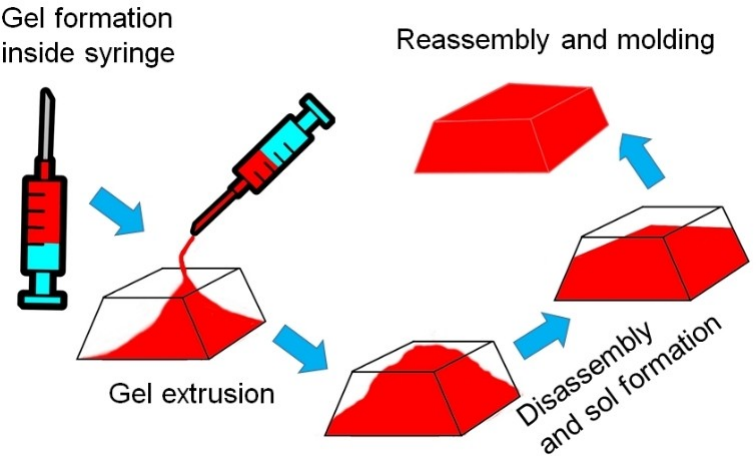
Demonstration of autonomous programming of homogeneous “molding and casting” of peptide hydrogel involving urease‐urea reaction.^[37]^ When an initially formed gel was extruded from the syringe, the extruded gel did not adapt to the shape of the container. With a gradual increase in pH, the gel changes from the initial distorted shape, producing a homogeneous solution inside the mold. With further time, regelation occurred, and a homogeneous gel was formed that conform to the shape of the mold.

We used the same enzymatic reaction to anneal pH‐responsive hydrogels (Figure 11).^[37]^ Annealing is applied for kinetically trapped systems to achieve thermodynamically more favourable equilibrium structures. Conventionally, a heat‐cool operation is performed to anneal gels,^[38]^ but can have disadvantages. We used a base‐triggered pH cycle to anneal the structures locally through uniform and control pH change under mild conditions combining the urea‐urease reaction and methyl formate; this resulted in a transient gel, the formation of a sol followed by reformation of a gel as the pH increased and then decreased again. We were able to prepare homogeneous and reproducible gels with a change in gel microstructures from spherulitic domains to a more uniform distribution of fibres. The changes in the fibre nature led to an increase in gel robustness. Control over the rate of pH change allowed us to control the properties of the annealed gel. There are two major advantages of our annealing approach than conventional annealing techniques. As it produced a free‐flowing solution in the intermediate stage, it was possible to use our annealing method for autonomous programming of homogeneous ‘molding and casting’ of the hydrogel assemblies in time. Additionally, this method could also be used to drive controlled mixing of encapsulated components within different gel environments.

We further modified the system by incorporating Ca^2+^ in the annealing process.^[25]^ At high pH, cross‐linking of the micellar dispersion of the carboxylate anions by Ca^2+^ resulted in formation of a Ca^2+^‐triggered gel; this prevented sol formation at the intermediate stage of annealing and maintained phase integrity throughout the pH increase and decrease. This is the first report of three stages of gel evolution in the literature. The mechanism of gel evolution thus mimics the concept of biological homeostasis. The preservation of the gel state (i. e., the phase integrity) throughout an energy cycle is the essential criterion of homeostasis.

**Figure 12:**
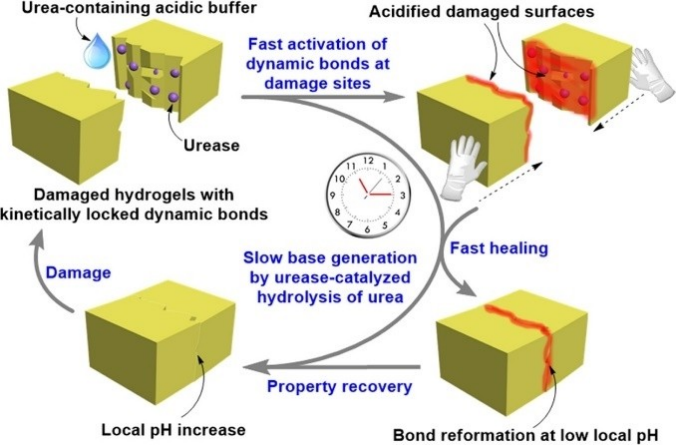
Representation of kinetically controlled damage‐healing experiment of hydrogel. A transient acidic pH state was temporarily created that allowed recovery of the damage with red colouration. The transient healability of damaged hydrogels was temporally programmed by combining a fast acidic activator (acylhydrazone activation) with the slow enzymatic generation of a base (urea‐urease reaction). Adapted with permission from Ref. [39]. Copyright © 2020, American Chemical Society.

Zhong *et al*. reported switching of a supramolecular polymeric hydrogel between two pH dependent states triggered by the urea‐urease reaction (Figure 12).^[39]^ The gel was fabricated by the in situ cross‐linking of acrylamide‐*co*‐diacetone acrylamide polymers with adipic acid dihydrazide. The chemical bond formation was faster at a low initial pH of 4 yielding a kinetically labile gel with a self‐healing ability. Increasing the pH to pH 7 resulted in a kinetically locked gel with no self‐healing properties. When the urease‐containing high pH hydrogel was damaged first, and then smeared with acidic buffer containing urea at the fracture surface, the acyl hydrogel bonds were activated due to protonation and the healing ability was restored. This enabled repair of the damage and restoration of the structures. Over time, the enzymatic reaction again drove the system to the high pH state with complete recovery of the material properties, especially the kinetic stability.

## 8 Current challenges and future perspectives

In spite of huge advantages of the urea‐urease reaction in controlling properties of pH responsive hydrogels, the method has several drawbacks. The major difficulty is that the enzyme losses activity at a pH of 3 or below. Hence, it needs precise optimization of reaction conditions so to maintain a starting pH near pH 3.5–4. Another issue is that the maximum pH that can be obtained cannot exceed the pH of 9.5.[^14^, ^20^] The presence of enzyme can also change the gel properties to some extent. We observed that presence of urease can result in a change in gel microstructures and reduce the gel stiffness.[^36^, ^37^] Although various reusable systems (like transient gels) can be synthesized involving the urease‐urea reaction, in practice there are eventual loss of enzyme activity in solution with time.^[19]^ Various sulfur‐containing compounds, polyphenols, quinones, hydroxamic acids etc. can also act as the enzyme inhibitor.[^6^, ^18^] George's group reported a twenty‐five times reduction in the growth of the fibres in presence of ethanolamine (3 equiv.) during supramolecular polymerization.^[26]^ Finally, the enzymatic reaction generates toxic ammonia which limits the gels in bio‐related applications without insulation.^[40]^

There are also certain aspects of this enzymatic reaction that are likely to be important but rarely investigated in gel chemistry. For example, the size of the reaction vessel. As the enzymatic reaction is associated with a pH dependent lag‐phase allowing the components to mix well in solution, ideally there should not be any change in the lag‐time as well as timescale for the pH jump if the size of the reaction vessel changes. It has been reported that in some aqueous‐organic interfaces like water with butanol, the presence of O_2_ increases the rate of interfacial inactivation of urease.^[24]^ Hence, for a large reaction vessel, the dissolved oxygen may influence the lag‐phase and thereby the temporal changes in assembly properties. Again, the atmosphere can be an issue here, and so the liquid or gel volume ratio to atmosphere can be a determining factor of the hydrogel properties. Moreover, carbon dioxide in the atmosphere can enter into the sample, resulting in the formation of carbonic acid or different carbonates. This can lead to local decrease of pH at the liquid/air interface, and thereby impose different rate of pH change or salt content at different layers of the solution.^[41]^ Hence, whilst a closed system (container inside which the gel is being formed) leads to homogeneous gel, conceptually an open system may produce an inhomogeneous gel with a pH gradient.

The urease‐urea reaction is extensively used in synthesizing photonic devices, biomaterials, sensors and actuators etc. composed of polymeric materials.[^40^, ^42^] In comparison, its use in the field of supramolecular gel is a recent trend. The pH dependent lag‐phase associated with the urea‐urease reaction provides advantage to control and adapt material properties. Most importantly, the dynamic behaviour of gels induced by the self‐regulating pH change allows one to produce materials that cannot be accessed directly. There is a huge scope in future to device gels with switchable and oscillating properties. The increasing popularity of this methods in gel chemistry certainly promises construction of new models of bioinspired functional gels.[^25^, ^35^]

## Conflict of interest

The authors declare no conflict of interest.

## Biographical Information

*Santanu Panja completed his B.Sc and M.Sc in Chemistry at the University of Calcutta, India in 2009 and 2011 respectively. In 2017, he received his Ph.D. degree from the University of Kalyani, India, under the supervision of Prof. Kumaresh Ghosh. He joined the University of Glasgow under Newton International Fellowship programme in March 2018 to work with Prof. Dave J. Adams. Presently he is associated with the same group as a research associate. His current research focuses on understanding the relationship between self‐assembly kinetics and the final properties of supramolecular gels*.

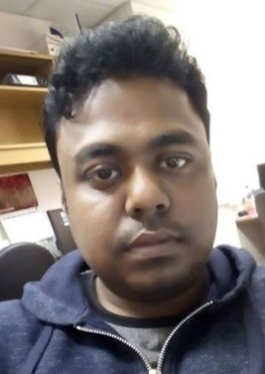

## Biographical Information

*Dave Adams carried out his PhD at the University of York. After postdoctoral work at the Universities of York, Leeds and Leicester, he joined Unilever R&D for 4 years. After this, he joined the University of Liverpool before moving to the University of Glasgow in 2016. His current research interests are in self‐assembly and soft materials*.